# Robotic assisted laparoscopic augmentation ileocystoplasty

**DOI:** 10.1590/S1677-5538.IBJU.2016.0205

**Published:** 2017

**Authors:** Peter A. Caputo, Daniel Ramirez, Matthew Maurice, Onder Kara, Ryan Nelson, Ercan Malkoc, Jihad Kaouk

**Affiliations:** 1 Department of Urology, Cleveland Clinic, Cleveland, Ohio, USA

## Abstract

**Introduction:**

Augmentation ileocystoplasty is a common treatment in adults with low capacity bladders due to neurogenic bladder dysfunction. We describe here our technique for robotic assisted laparoscopic augmentation ileocystoplasty in an adult with a low capacity bladder due to neurogenic bladder dysfunction.

**Materials and Methods:**

The patient is a 35 years-old man with neurogenic bladder due to a C6 spinal cord injury in 2004. Cystometrogram shows a maximum capacity of 96cc and Pdet at maximum capacity of 97cmH_2_O. He manages his bladder with intermittent catheterization and experiences multiple episodes of incontinence between catheterizations. He experiences severe autonomic dysreflexia symptoms with indwelling urethral catheter. He has previously failed non operative management options of his bladder dysfunction. Our surgical technique utilizes 6 trocars, of note a 12mm assistant trocar is placed 1cm superior to the pubic symphysis, and this trocar is solely used to pass a laparoscopic stapler to facilitate the excision of the ileal segment and the enteric anastomosis. Surgical steps include: development of the space of Retzius/dropping the bladder; opening the bladder from the anterior to posterior bladder neck; excision of a segment of ileum; enteric anastomosis; detubularizing the ileal segment; suturing the ileal segment to the incised bladder edge.

**Results:**

The surgery had no intraoperative complications. Operative time was 286 minutes (4.8 hours). Estimated blood loss was 50cc. Length of hospital stay was 8 days. He did experience a postoperative complication on hospital day 3 of hematemesis, which did not require blood transfusion. Cystometrogram at 22 days post operatively showed a maximum bladder capacity of 165cc with a Pdet at maximum capacity of 10cmH_2_O.

**Conclusions:**

As surgeon comfort and experience with robotic assisted surgery grows, robotic surgery can successfully be applied to less frequently performed procedures. In this case we successfully performed a robotic assisted laparoscopic augmentation ileocystoplasty displaying improvement in measurable functional outcomes.

## ARTICLE INFO

**Available at: http://www.intbrazjurol.com.br/video-section/20160205_caputo_et_al**

**Int Braz J Urol. 2017; 43 (Video #13): 994**

